# A rosin-functionalized plastic surface inactivates African swine fever virus

**DOI:** 10.3389/fvets.2024.1441697

**Published:** 2024-09-23

**Authors:** Johanneke Dinie Hemmink, Sailee Shroff, Naomi Chege, Marjo Haapakoski, Linda K. Dixon, Varpu Marjomäki

**Affiliations:** ^1^Animal and Human Health Program, International Livestock Research Institute, Nairobi, Kenya; ^2^Department of Biological and Environmental Sciences/Nanoscience Center, University of Jyväskylä, Jyväskylä, Finland; ^3^African Swine Fever Virus Group, The Pirbright Institute, Pirbright, United Kingdom

**Keywords:** African swine fever, ASFV, rosin, antiviral surface, plastic

## Abstract

African swine fever virus (ASFV) causes a severe hemorrhagic disease in pigs, leading to up to 100% case fatality. The virus May persist on solid surfaces for long periods; thus, fomites, such as contaminated clothing, footwear, farming tools, equipment, and transport vehicles, May contribute to the indirect transmission of the virus. Here, a plastic surface functionalized with tall oil rosin was tested against ASFV. The rosin-functionalized plastic reduced ASFV infectious virus titers by 1.3 log_10_ after 60 min of contact time and killed all detectable viruses after 120 min, leading to a ~ 6 log_10_ reduction. In contrast, the infectious virus titer of ASFV in contact with low-density polyethylene (LDPE) plastic reduced <1 log_10_ after 120 min. Transmission electron microscopy (TEM) showed significant morphological changes in the virus after 2 h of contact with the rosin-functionalized plastic surface, but no changes were observed with the LDPE plastic. The use of antiviral plastic in the farming sector could reduce the spread of ASFV through fomites and could thus be part of an integrated program to control ASFV.

## 1 Introduction

African swine fever (ASF) is a severe hemorrhagic disease in pigs, leading to up to 100% fatality in infected animals. The disease is caused by the ASFV, which is transmitted through direct contact with infected domestic or wild pigs, pig products, or fomites. Fomites are objects or materials that are likely to carry infection. This could include all objects and materials that come into contact with infected pigs, such as feeds, housing, clothes, footwear, equipment, and transport vehicles (1). In addition, in sub-Saharan Africa, there is evidence for the presence of a sylvatic cycle, where ASFV is transmitted between warthogs and soft ticks of the *Ornithodoros* genus (2). In most countries in Sub-Saharan Africa, the disease is endemic, and several genotypes May circulate in an area simultaneously (3). Outside of Africa, only genotypes I and II, as well as recombinants of these genotypes, have been detected. ASFV genotype II spread to the Caucasus region of Georgia in 2007 and has since spread to many other countries in Europe, Asia, Oceania, and the Caribbean (4). Thus, ASFV poses a threat to the pork industry globally. In the absence of treatment and globally available effective vaccines, biosecurity measures are essential to prevent the spread of the disease. Biosecurity measures include limiting the movement of animals, people, and materials/equipment between farms, using foot baths, providing farm-specific PPE, and disinfecting equipment and vehicles entering and leaving the farm (5).

ASFV is a large and complex double-stranded DNA virus, which consists of multiple concentric layers and has a diameter of approximately 260–300 nm. The outermost layer is the external envelope, which is derived from the cellular plasma membrane during the budding process by which the virus egresses from the cell. Interior to this, the virus has an icosahedral outer capsid with a diameter of approximately 200 nm. Underneath the outer capsid lies an inner membrane, which surrounds the core shell and the nucleoid. Both the intracellular virion, without the outer envelope, and the extracellular virion, with the outer envelope, are infective *in vitro* (6–9).

The ASF virus is inactivated by treatment at 60°C for 30 min but remains infectious at lower temperatures and when stored frozen over extended periods. It has been estimated that ASFV can remain infectious for 8.5 or 15.3 days at 4°C, respectively (10). Furthermore, viable viruses can remain on the tissues of pig carcasses for several months, especially at low temperatures (11). Some studies have investigated ASFV viral survival in feed, bedding materials, and soil. Stoain et al. (12) demonstrated that the half-life of ASFV varied from 9.6 to 14.2 days when viral survival was studied in nine different feed ingredients in conditions that simulated trans-Atlantic shipment. Field viruses could be isolated from bedding materials for some days to weeks at 4°C, with the longest survival in bark. However, in soil, the stability of ASFV was shown to vary from a few days up to several weeks (13, 14). In rivers, the water virus remained infectious when maintained at 4°C but was inactivated after 28 or 14 days when maintained at 15 or 21°C (15). In samples stored between +4 and + 23°C, the inactivation of the virus was faster in water, soil, and leaf litter compared to straw, hay, and grain. In a study by Petrini et al. (16), infectious ASFV was detected in dry-cured meat products (loin and pork belly) prepared from infected pigs even after 2 months from the beginning of the processing.

Coniferous resin acids are known to have antimicrobial properties against a range of pathogens, including Gram-positive bacteria, fungi, and enveloped viruses (17–21). Rosin is a by-product of the forest industry and contains resin acids. We found recently that rosin-functionalized plastic leached the active component to the surface of the plastic and very potently reduced the infectivity of SARS-CoV-2 and seasonal coronavirus (HCoV-OC43) (19). This study aimed to investigate whether rosin-functionalized plastic has antiviral activity against the structurally complex ASFV.

## 2 Materials and methods

### 2.1 Plastic material

Two types of plastic surfaces were used for our study: low-density polyethylene (standard LDPE) and rosin-functionalized plastic (PREXELENT^®^), generously provided by Premix Ltd. PREXELENT^®^ is a functional plastic that is prepared by incorporating 10 wt-% of rosin into LDPE (patent WO2018229190A1). The plastic surfaces were 1 cm^2^.

### 2.2 Surface antiviral test

To test the contact time required for ASFV to be inactivated by the rosin-functionalized plastic (PREXELENT^®^) or LDPE plastic, ASFV was added to the plastics in triplicate and incubated for 5, 10, 30, 60, 120, 240, or 480 min. Before starting, the plastics were sterilized by immersing them in 70% ethanol for 30 s and wiping them with sterile tissue paper. They were then left to dry out and transferred to 24-well plates with 6 mL sterile water between the wells to prevent drying out of the plastics and incubated at 37 ⁰C overnight. On the day of the assay, a volume of 50 μL of ASFV_Kenya1033 was used for each replicate (corresponding to 1×10^6^ HAD_50_). ASFV_Kenya1033 is a genotype IX ASFV and was grown in primary porcine macrophages (22). A volume of 50 μL ASFV_Kenya1033 was added on top of triplicate pieces of each plastic for each incubation time (5, 10, 30, 60, 120, 240, and 480 min). After incubation at room temperature (20–25°C) for a set amount of time, 450 μL of complete RPMI [RPMI 1640 (Sigma Aldrich, UK), supplemented with 2 mM L-glutamine (Sigma Aldrich, UK), 10% fetal bovine serum, 100 UI/mL of penicillin (Sigma Aldrich), and 100 mg/mL of streptomycin (Sigma Aldrich, UK)] was added to each of the wells. The plates were rocked on a plate shaker for exactly 1 min, and the resulting media was collected and aliquoted for ASFV quantification. For DNA extraction and real-time quantitative PCR analysis (qPCR), a volume of 200 μL was aliquoted and the remainder volume was aliquoted for analysis by hemadsorption assay_._

### 2.3 Quantitative determination of ASFV

The ASFV genome copy number in samples was determined by amplification of p72/B646L using a qPCR assay adapted from King et al. (23). DNA was extracted from 200 μL of the sample using the DNeasy^®^ Blood & Tissue Kit (Qiagen) according to the manufacturer’s protocol. The resulting DNA was used for the qPCR analysis with primer and probe sequences adapted to the ASFV_Kenya1033 strain. The primer sequences were P72-F (5’-CTGCTCACGGTATCAATCTTATCGA-3′) and P72-R (5’-GATACCACAAGATCAGCCGT-3′), and the P72 probe was 5’-FAM-CCACGGGAGGAATACCAACCCAGCG-TAMRA-3′. Each reaction was conducted in duplicates in 20 μL of reaction mixture containing 6.5 μL of nuclease-free water, 10 μL of TaqMan Fast Advanced master mix (Applied Biosystems), 0.6 μL of forward primer (10 μM), 0.6 μL of reverse primer (10 μM), 0.3 μL of TaqMan probe (10 μM), and 2 μL of template DNA. The plasmid standard was diluted 10-fold from 10^9^ to 10^1^ copies/ well. Nuclease-free water was used as no template control.

The titer of infectious virus in samples was determined using a hemadsorption assay. The samples were 10-fold serial diluted in complete RPMI (see above), and the different dilutions were used to infect porcine pulmonary alveolar macrophages (PAMs) in 96-well plates using four replicates per dilution for each sample. The next day, red blood cells were added to the plates (1:200 diluted in PBS). The plates were examined for the presence of the ASFV characteristic rosettes using an inverted microscope on day 5. The titers of infectious viruses in the samples were calculated using the Reed and Muench method.

### 2.4 Preparation of virus for imaging

ASFV (genotype II ASFV grown in primary porcine macrophages; 1×10^6^ HAD_50_) was added onto the surface of the plastic surfaces that were placed in a well of a 12-well tissue culture plate and sealed. The virus was added in 10 μL of the stock virus, and a 13 mm coverslip was added to ensure good contact for 2 h incubation time at room temperature. After 2 h, 500 μL of 10% formaldehyde fixative was added, which lifted the coverslip off. The coverslip was then taken gently away, and fixative incubation continued for 48 h at room temperature. The fixative solution containing detached viruses was transferred to tubes and fresh 10% formaldehyde fixative was added to cover the plastic surface. The plastic sheet and fixative containing detached viruses were placed in containers for shipment to Finland.

### 2.5 Imaging of virus using transmission electron microscopy

To concentrate the sample for electron microscopy, 350 μL of the media that was flushed from the rosin-functionalized plastic (PREXELENT^®^) and LDPE plastic was centrifuged gently using the Beckman Coulter Airfuge™ centrifuge (20 psi, 30 min, A-95 rotor). The pelleted virus was gently dissolved in a small volume of 10–15 μL of the supernatant left in the tube after pelleting.

For the TEM sample preparation, 5 μL of the pelleted virus was applied to glow-discharged Butvar-coated copper grids (EMS/SC7620 mini-sputter coater) for 15 s. Thereafter, the excess sample was blotted away using blotting paper (Whatman 3MM). The samples were negatively stained with 1% phosphotungstic acid for 10 s, and the excess stain was blotted away as mentioned before. The samples were airdried overnight and imaged using the JEOL JEM-1400 transmission electron microscope (JEOL, Tokyo, Japan) equipped with a LaB_6_ filament.

### 2.6 Statistical analysis

Statistical analysis was performed using GraphPad Prism6 software. Significant differences between groups were determined using a t-test for each time point, adjusted for multiple comparisons.

## 3 Results

### 3.1 Rosin-functionalized plastic reduces ASFV infectivity

To establish whether rosin-functionalized plastic had an antiviral effect against ASFV, ASFV was incubated with the rosin-functionalized plastic or LDPE plastic for different lengths of time (5, 10, 30, 60, 120, 240, and 480 min). The resulting virus was washed with complete RPMI, and the viral titers were determined using qPCR and HAD_50_ assay.

**Figure 1:**
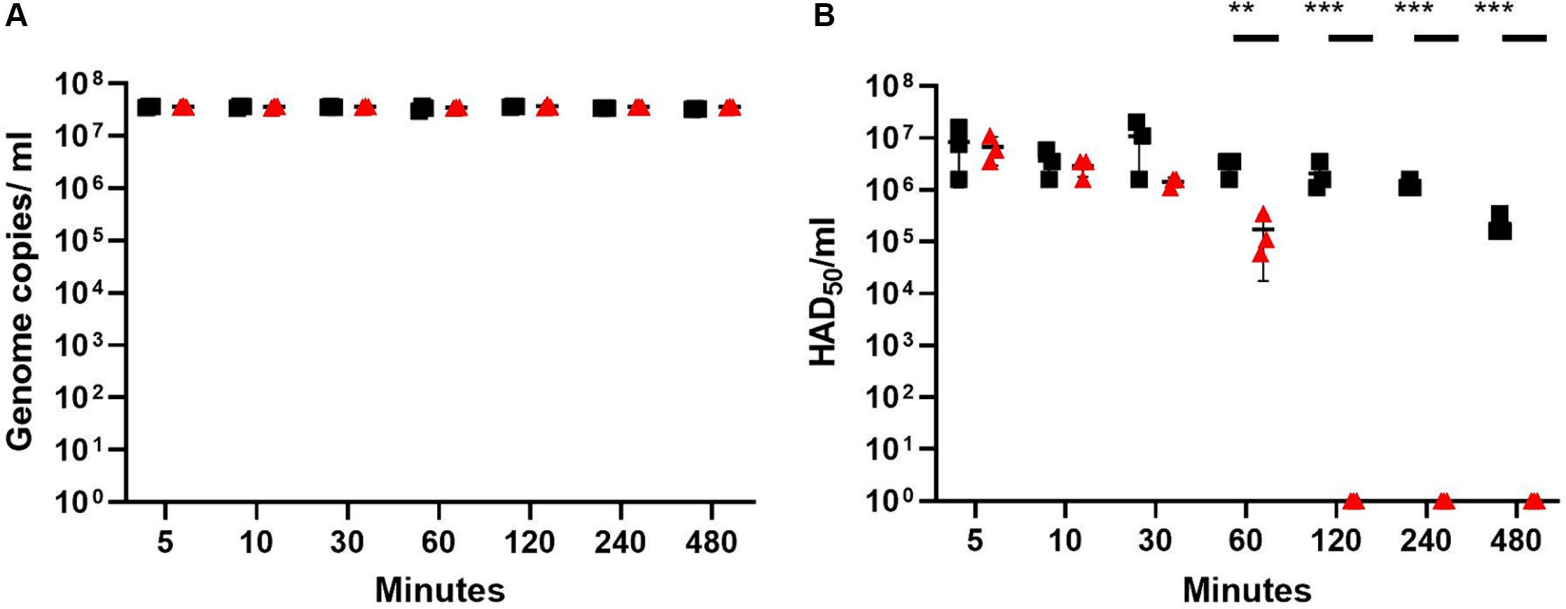
ASFV titers after contact with rosin-functionalized (PREXELENT^®^) or LDPE plastic as determined using qPCR **(A)** or HAD_50_ assay **(B)**. ASFV at x10^6^ HAD_50_ was incubated with rosin-functionalized plastic (PREXELENT^®^) (red triangles) or LDPE plastic (black squares) for 5, 10, 30, 60, 120, 240, or 480 min and flushed with RPMI. ASFV genome copies from the flush were determined by qPCR **(A)** and viable virus titers by HAD_50_ assay **(B)**. Statistical significance was determined using t-tests for each time point, adjusted for multiple tests (**p < 0.01, *** *p* < 0.001).

For all the time points, the number of genome copies detected by qPCR in the flushed-out medium remained constant across the different time points for both plastics, indicating that the virus does not strongly adhere to either of the plastics (Figure 1A). In contrast, the HAD_50_ assay, which determines the titer of viable virus, showed that the viral titer was reduced by 1.3 log_10_ after 60 min of incubation with the rosin-functionalized plastic. Strikingly, after 120-, 240-, and 480-min incubation, no red blood cell rosettes were detected using the HAD_50_ assay for the virus incubated with the rosin-functionalized plastic, confirming a ~ 6 log_10_ reduction for these time points (Figure 1B). In contrast, the viable virus titer of the virus in contact with LDPE plastic was reduced by <1 log_10_ after 120 min after incubation. The viability of the PAMs used for the HAD_50_ assay was not affected by the flushed medium from either plastic after 4 h of incubation (data not shown). These results suggest that the rosin-functionalized plastic was very efficient in reducing the infectivity of ASFV on its surface.

### 3.2 Rosin-functionalized plastic affects ASFV morphology

**Figure 2:**
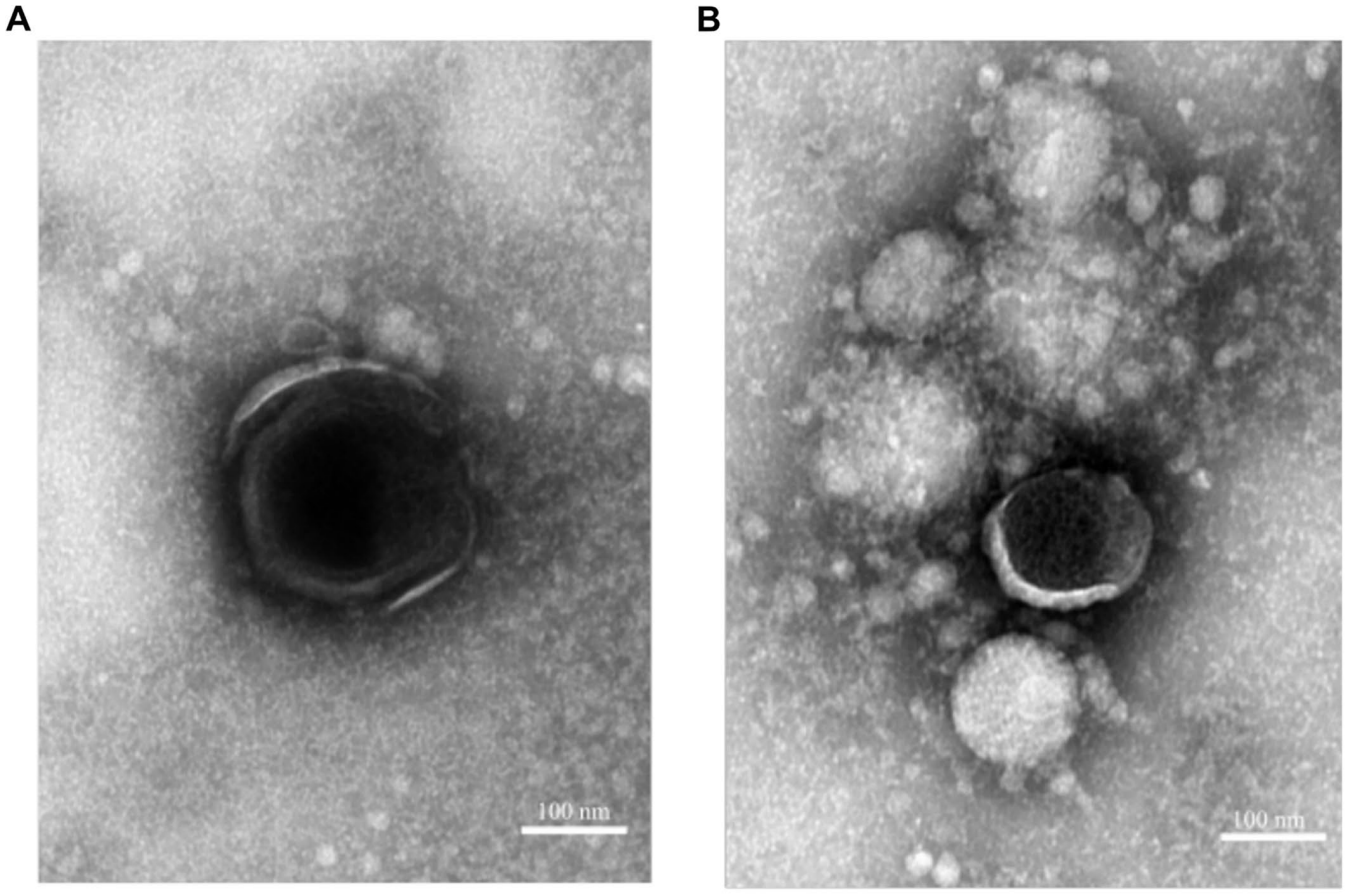
ASFV as visualized by TEM incubated with rosin-functionalized plastic (PREXELENT^®^) **(A)** or LDPE plastic **(B)**. Viruses were gently pelleted down from fixative solution and subjected to negative staining (see Material and Methods section). Bar, 100 nm.

To investigate the effect of incubation with rosin-functionalized plastic on the virus structure, TEM was performed. ASFV was added to the plastic surfaces for 2 h at 37°C. Thereafter, a fixative solution was added directly to the surface for an extended period of time. As the samples were then shipped from the Pirbright Institute to the University of Jyväskylä, Finland, it was likely that many of the viruses had detached from the surface to the fixative solution. Furthermore, we found previously that ASFV was easily detached from the plastic surfaces (Figure 1A). Therefore, the imaging of the viruses was carried out using the fixative solution collected from the plastic samples. After gently sedimenting the viruses using an Airfuge for 30 min, the samples were applied onto TEM grids and negatively stained. Observations under an electron microscope demonstrated that only intact-looking virions (10) were found in the solution from the LPDE plastic samples (Figure 2A). In contrast, we could not find any intact virions from the samples flushed from the rosin-functionalized plastic, suggesting that the majority of the ASFV had undergone significant morphological changes on the rosin-functionalized plastic surface (Figure 2B). These results, together with infectivity measurements, demonstrate efficient antiviral action of the rosin-functionalized plastic within a 2-h time frame.

## 4 Discussion

The resin acid family covers a spectrum of antimicrobial activities against several micro-organisms, including Gram-positive bacteria, enveloped viruses and fungi (17, 18, 20, 21, 24). Although the mechanism of action is not known, due to the differences in effectivity against Gram-positive bacteria and Gram-negative bacteria, it was hypothesized that differences in the cell membranes are important for the mechanism of action of rosin and resin acids against Gram-positive bacteria (21, 24). Rosin-containing soap also showed virucidal activity against enveloped viruses in 5 min (e.g., influenza A virus, respiratory syncytial virus, and SARS-CoV-2), but failed to affect the non-enveloped encephalomyocarditis virus. The authors thus suggested that the target for the antiviral activity of rosin soap is the viral envelope (17). ASFV is infective *in vitro* with or without the outer envelope and thus it was of interest to investigate whether rosin-functionalized plastic has an effect against this virus.

Indeed, the rosin-functionalized plastic exhibited an antiviral effect against ASFV, starting from 60 min of contact time and achieving complete inactivation after 120 min of contact. Previously, it was shown that the rosin-functionalized plastic efficiently inactivates coronaviruses already after 15 min of contact (19). Even though a much longer contact time is required to inactivate ASFV, the antiviral activity against ASFV in this study demonstrates that the rosin-functionalized plastic also acts against more complex viruses.

Schroff et al. showed that contact with rosin-functionalized plastic did not lead to apparent structural changes in SARS-COV-2 and seasonal human coronavirus. Although the exact mechanism of action was not elucidated, the viruses were able to bind the host cell surface and enter the endosomes. However, the block in infectivity occurred in the endosomal membrane fusion step between the viral envelope and the endosomal membranes (19). In contrast, TEM imaging performed in this study showed changes in the morphology of ASFV after 2 h of contact with rosin-functionalized plastic. Even though we did not investigate the ability of the virus to bind and enter the host cell, we hypothesize rosin might have different modes of action against enveloped viruses. It would be interesting to investigate the mechanism of action against ASFV in future studies. It would also be interesting to know whether the rosin-functionalized plastic has antiviral activity against other livestock pathogenic viruses.

Rosin-functionalized plastics have promising applications in the livestock sector, given any object or material in contact with infected animals could act as a fomite for virus transmission. The tested functionalized plastic belongs to the low-cost commodity plastics (LDPE) and shows great durability typical of these plastics. As the plastic May contain a high amount of effective rosin (e.g., 10%), it May remain active for months to years. With the demonstration of antiviral activity against ASFV, a very stable virus, the rosin-functionalized plastic is likely to exhibit antiviral effects against a broad spectrum of livestock pathogenic viruses. Consequently, incorporating rosin-functionalized plastic to diminish viable pathogen loads in fomites could contribute to the reduction of pathogen transmission within and between farms. Carlson et al. (13) demonstrated that the virus survival was largely dependent on soil structure and pH; sandy soil was more optimal for viral stability compared to acidic forest soil. Therefore, it would need to be validated whether the rosin-functionalized plastic is still effective when organic materials are present and/or when cleaning agents and disinfectants are used. This could limit the applicability of the rosin-functionalized plastics to areas or items with minimal organic contamination. The use of effective antiviral surfaces could play a pivotal role in integrated control programs aimed at mitigating infectious diseases. Examples of potential applications on the farm could include door handles, workbenches, tools, and toolboxes used by farm workers and visitors. The plastic May also be applicable in laboratories handling samples potentially containing viruses.

## Data Availability

The original contributions presented in the study are included in the article/supplementary material, further inquiries can be directed to the corresponding author.
